# Study on potential invasion threat area of *Nicotiana glauca* Graham based on the MaxEnt model

**DOI:** 10.3389/fpls.2026.1796997

**Published:** 2026-05-21

**Authors:** Mengna Xu, Ensen Guan, Dahai Wang, Shujin Cheng, Hua Yu

**Affiliations:** 1 College of Plant Protection, Shandong Agricultural University, Tai’an, China; 2 Luozhuang Experimental Station, Shandong Weifang Tobacco Co., Ltd., Weifang, China; 3 Production Management Department, General Tobacco Group Co., Ltd., Jinan, China

**Keywords:** ecological niche, invasion threat area, invasive (exotic non-native) species, MaxEnt, *Nicotiana glauca* Graham

## Abstract

*Nicotiana glauca* Graham (*N. glauca)* is an agricultural weed, environmental weed, and noxious weed, posing a significant threat to ecosystems and agricultural systems. To provide a basis for the early warning of *N. glauca*, the existing geographical distribution data and environmental variables of *N. glauca* were used to predict the potential invasion threat area under historical (1970-2000) and future (2061-2080) climate conditions through MaxEnt and ArcGIS in this study. The results showed that the isothermality (bio3), the max temperature of warmest month (bio5), the mean temperature of wettest quarter (bio8), and the mean diurnal range (bio2) were the key bioclimatic variables affecting the growth of *N. glauca*. The potential invasion threat area of *N. glauca* under historical climate conditions was mainly distributed in the central and southern parts of North America, the vast majority of South America, the northern coastal areas and the central and southern parts of Africa, the western and southern parts of Europe, the central and southern parts of Asia, and the vast majority of Oceania, among which the high-threat areas were mainly located in the southern regions of each land. Among the four scenarios of future climate, the centroid of the invasion threat area shifted southeastward and northeastward, and the total area of the invasion threat area was larger than that under historical climate conditions, suggesting the arduousness of the task of preventing the invasion of *N. glauca*. The results of this study provided valuable information and theoretical references for the early warning of *N. glauca*.

## 1 Introduction

Invasive species, also referred to as “transformative species”, which means they can change the character, condition, and other attributes of natural ecosystems across a substantial area (Richardson et al., 2000). They can disrupt the structure and function of ecosystems, impair species diversity, and cause economic losses. Currently, most countries worldwide are severely affected by invasive species. Up to now, the number of documented alien invasive plant species alone in China has surpassed 520 (Cui et al., 2025). The invasion of alien species is closely intertwined with global climate change, with mutual interactions between them. Therefore, assessing the invasion risk and predicting the distribution of invasive species holds great significance for their prevention, early warning, and scientific management.

*Nicotiana glauca* Graham (*N. glauca*), an invasive plant species native to Argentina, Bolivia, Paraguay, and Uruguay in South America (Dounas et al., 2023; Ollerton et al., 2012), belongs to the genus *Nicotiana* of the Solanaceae family. It has been listed in the Global Compendium of Weeds (GCW) as an agricultural weed, negligently introduced exotic species, weed escaped from planting areas, environmental weed, and noxious weed. *N. glauca* can grow up to 6 meters in height and produces a large number of tiny seeds, which can be dispersed by wind and water, and the entire plant is toxic to humans, animals and plants (Furer et al., 2011; Robert et al., 2010). Previous studies have revealed that *N. glauca* can invade semi-natural or natural habitats of conservation value, threatening their pristine environments and even affecting the life activities of wild animals (Cronk and Fuller, 2001). Specifically, a study on the invasion of *N. glauca* in the Taif region, Western Saudi Arabia, found that its invasive activities inhibit the growth of most local species. Meanwhile, while adapting to the local soil structure, *N. glauca* potentially modifies the soil properties (Assaeed et al., 2021). Specifically, some studies have revealed that *N. glauca* alters the soil microbial community composition and its carbon cycle-related functions in semi-arid Mediterranean environments (Rodríguez-Caballero et al., 2020). In China, *N. glauca* is cultivated in Tianjin, Hebei, and other regions due to its medicinal value. However, its toxicity and invasiveness cannot be ignored. Furthermore, the cuticular wax of *N. glauca* contributes to its adaptation to arid environments (Negin et al., 2023). Yet, there remains a lack of systematic investigation into the distribution of its potential invasive areas at present. Therefore, it is of particular necessity to monitor and predict the global suitable distribution areas of *N. glauca*.

Common modeling methods for species distribution prediction include various algorithms, such as the Maximum Entropy model (MaxEnt), Generalized Linear Model (GLM), and genetic algorithm for rule set production (GARP) (Sandhya Kiran et al., 2024). Among them, MaxEnt has the advantages of small influence by sample size, fast calculation speed and high operation efficiency, and has been used in the study of potential suitable habitats of invasive alien species such as *Amaranthus palmeri* S. Watson (Zhang et al., 2022), *Cenchrus spinifex* Cav (Cao et al., 2021), *Solanum rostratum* Dunal (Huang et al., 2024), *Spartina alterniflora* Loisel (Yuan et al., 2021), and *Emex australis* (Quan et al., 2023). The existing distribution data of *N. glauca* exhibit regional aggregation, with dense samples in South America and sparse samples in Asia. The application of MaxEnt can effectively mitigate the impact of sample bias.

Therefore, MaxEnt was employed in this study to explore the potential invasion areas of *N. glauca* under historical (1970–2000) and future (2061–2080) climatic conditions. The results will provide a theoretical reference for the scientific prevention, control, and management of *N. glauca* and contribute to the sustainable management of resources.

## 2 Materials and methods

### 2.1 Acquisition of sample records *of N. glauca*

A total of 27,451 extant sample records of *N*. *glauca* were collected from the Global Biodiversity Information Facility (GBIF, https://www.GBIF.org) and online literature in this study. 25,549 sample records were obtained by removing duplicate data and incomplete data lacking of longitude and latitude before analysis.

### 2.2 Acquisition of bioclimatic variables

Nineteen bioclimatic variables were downloaded from the WorldClim Global Climate Database (version 2.1) (http://www.worldclim.org) for historical global climate conditions with a spatial resolution of 2.5 min. These variables record climate averages for the years 1970–2000 and include annual trends (mean annual temperature and precipitation), seasonality (seasonal range of temperature and precipitation), and extreme environmental factors (extreme temperature and rainfall) (**Table 1**).

**Table 1:** Nineteen bioclimatic variables.

Bioclimatic variables	Variable description	Unit
bio1	Annual Mean Temperature	°C
bio2	Mean Diurnal Range	°C
bio3	Isothermality	—
bio4	Temperature Seasonality	—
bio5	Max Temperature of Warmest Month	°C
bio6	Min Temperature of Coldest Month	°C
bio7	Temperature Annual Range	°C
bio8	Mean Temperature of Wettest Quarter	°C
bio9	Mean Temperature of Driest Quarter	°C
bio10	Mean Temperature of Warmest Quarter	°C
bio11	Mean Temperature of Coldest Quarter	°C
bio12	Annual Precipitation	mm
bio13	Precipitation of Wettest Month	mm
bio14	Precipitation of Driest Month	mm
bio15	Precipitation Seasonality	—
bio16	Precipitation of Wettest Quarter	mm
bio17	Precipitation of Driest Quarter	mm
bio18	Precipitation of Warmest Quarter	mm
bio19	Precipitation of Coldest Quarter	mm

To predict the future global invasive areas of *N. glauca*, four shared socio-economic pathways (SSPs), SSP1-2.6, SSP2-4.5, SSP3-7.0, and SSP5-8.5 (Adhikari et al., 2025), were adopted from the moderate resolution National Climate Center climate system model (BCC-CSM2-MR) for the year 2070 (2061–2080 average) of the Coupled Model Intercomparison Project Phase 6 (CMIP6) (BCC, 2019; Wu et al., 2019). SSP1-2.6 is the sustainable development pathway with warming limited to less than 2 °C, SSP2-4.5 is a moderate development path with warming limited to less than 3 °C, SSP3-7.0 is the partial development pathway with warming limited to less than 4.1 °C, and SSP5-8.5 is the conventional development path with warming limited to less than 5 °C.

### 2.3 Processing of sample records *of N*. *glauca*

**Figure 1:**
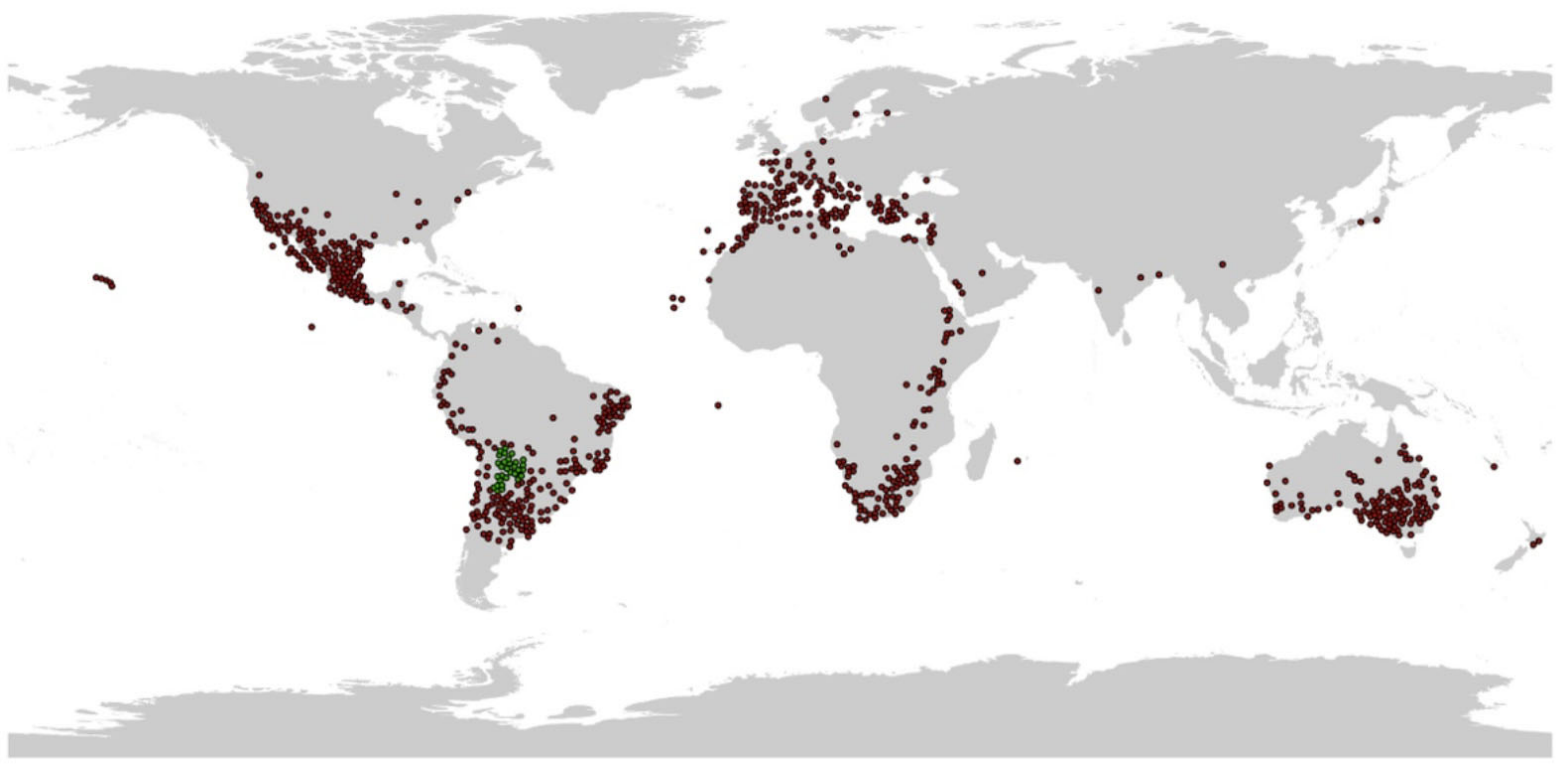
Sample records of *N. glauca*. Native (green, 33 dots) and invaded (red) dots, the obtained 759 sample records in total. The grey world map was obtained from WorldClim (https://worldclim.org), and this figure was drawn with ArcGIS 10.8.

Species sample records are usually collected non-randomly, and some of the records are highly similar in location, which can lead to overfitting of the model and have an impact on the model results. The data were sparsified using the spThin package (spatial thinning function for species occurrence records) in the R Studio software, where the data information was converted to.csv format, and the thin.par thinning parameter was set to 100 km. A thinning distance of 100 km was employed to eliminate redundant records, mitigate spatial autocorrelation among occurrence points, and match the global geographic scope of this study. Furthermore, this distance avoids the excessive loss of sample points associated with finer-scale thinning thresholds (Nyarko and Bayor, 2018). And the number of repetitive refinements of the reps was 500. Then the obtained coordinate records were sparsified by selecting a random point from each of the sample cells that contained one or more sample records. Finally, 759 sample records were obtained (**Figure 1**).

### 2.4 Screening and pre-processing of bioclimatic variables

MaxEnt modeling software downloaded from https://biodiversityinformatics.amnh.org/open_source/maxent/, was used for maximum entropy simulation to predict the potential invasion threat area of *N. glauca*. To avoid overfitting due to covariance among bioclimatic variables, the sample records of *N. glauca* and 19 bioclimatic variables were first pre-simulated in MaxEnt to derive the contribution of each bioclimatic variable, and then Pearson correlation analysis was performed using SPSS software. After that, based on the pre-simulation experiment and correlation analysis, bioclimatic variables with a contribution less than 1% and a correlation coefficient more than 0.7 were removed. Finally, six bioclimatic variables were obtained (**Table 2**), which were used as the dominant environmental factors to predict the potential invasion threat area of *N. glauca*. *N. glauca* favors drier, less seasonal, and thermally stable environments (Issaly et al., 2024). These six selected variables had high ecological relevance: bio2 reflects diurnal temperature fluctuation which related to the species’ broad elevational tolerance; bio3 measures temperature stability, aligning with its preference for less seasonal climates; bio5 defines the upper thermal tolerance limit for this hot-region native; bio8 represents water-temperature interaction during a key phenological phase (germination/establishment); bio15 quantifies moisture variability with low values indicating preferred stable moisture regimes; bio18 captures growing-season moisture supply, critical in arid environments. These variables were selected for their direct ecological interpretability in characterizing the thermal limits, seasonal stability, and water availability defining the fundamental niche of *N. glauca*. Taken together, these variables are all directly ecologically interpretable and can characterize thermal limits, seasonal stability, and water availability to determine the basic niche of bluegrass.

**Table 2:** Bioclimatic variables used for the MaxEnt model.

Bioclimatic variables	Variable description	Unit
bio2	Mean Diurnal Range	°C
bio3	Isothermality	—
bio5	Max Temperature of Warmest Month	°C
bio8	Mean Temperature of Wettest Quarter	°C
bio15	Precipitation Seasonality	—
bio18	Precipitation of Warmest Quarter	mm

### 2.5 Optimizing parameters of the MaxEnt model

Key factors influencing the complexity and prediction accuracy of the MaxEnt model include the Regularization Multiplier (Gonzalez et al.) and Feature Combination (FC). The FC consists of five features: linear (L), quadratic (Q), hinge (H), product (P), and threshold (T). To avoid the risk of overfitting, we utilized the ENMeval package in R for parameter optimization, selecting four feature combinations: LQ, LQH, LQHP, and LQHPT. We calculated the minimum deltaAICc value and applied it to the model. The results showed that setting RM to 1 and FC to LQHP was the optimal parameter combination for the model in this study.

### 2.6 Parameter settings and model simulation of the MaxEnt model

The obtained 759 occurrence records of *N*. *glauca* and bioclimatic variables were imported into the MaxEnt model to carry out the training and testing work of the MaxEnt model. 75% of the randomly selected sample records were trained and the remaining 25% were tested. The regularization multiplier was 1, the output file format was asc, and the type was logistic. Random seed was selected randomly, i.e., a different random seed was used for each run. The number of iterations was set to 10 in order to cut down on uncertainty caused by outliers, and the number of replicates was set to 10. The maximum number of iterations was 10,000. To prevent the model predictions from being too high or too low, RM was set to 1, and FC was set to LQHP.

For the prediction under future climate conditions (2061-2080), the corresponding air layer data for SSP1-2.6, SSP2-4.5, SSP3-7.0, and SSP5-8.5 were imported into the projection layer directories of the MaxEnt model, and other parameters were consistent with the settings under historical climate conditions.

### 2.7 MaxEnt model performance evaluation

The subject operating characteristic curve (Issaly et al.) is a well-recognized metric for evaluating the model performance of MaxEnt. Model accuracy was analyzed using the area under the ROC curve (i.e., AUC), which takes values from 0 to 1. When 0<AUC ≤ 0.6, the prediction is a failure. When 0.6<AUC ≤ 0.7, the prediction accuracy is low. When 0.7<AUC ≤ 0.8, the prediction accuracy is general. When 0.8<AUC ≤ 0.9, the prediction accuracy is good. When 0.9<AUC ≤ 1.0, the prediction accuracy is very good. The True Skill Statistic (TSS), Continuous Boyce Index (CBI) and Kappa were also used to evaluate the accuracy of the MaxEnt model. Their values all range from -1 to 1, with higher positive values indicating better model performance.

### 2.8 Classifications of the invasion threat area

ArcGIS 10.8 was used for layer analysis, data sparsification, data format transformation, and reclassification of results. The Natural Breaks Method was used to classify the level of invasion threat in the area. The output file (ASCII) of MaxEnt was converted to raster format using Arc Toolbox, a toolkit in ArcGIS. Then the probability layer (0-1) of the invasion threat area was classified into different levels using reclassification in the spatial analysis tool. The occurrence probability values output by MaxEnt generally exhibit a non-uniform distribution. The Natural Breaks Method can identify breakpoints in the dataset, maximizing within-class similarity and between-class dissimilarity. This method is easy to implement, reflects the actual distribution of values more realistically than the equal interval method, and is also a commonly used classification approach in ArcGIS. Therefore, Natural Breaks Method was used to classify the invasion threat area of *N*. *glauca* into four grades: non-threat (<0.09), low-threat (0.09-0.26), medium-threat (0.26-0.47), and high-threat (>0.47), to produce a map of the potential invasion threat area of *N*. *glauca*. In fact, the prediction result of MaxEnt should accurately be the suitable area of *N*. *glauca*. We approximately regarded the suitability degree as the degree of invasion risk, and simply believed that high suitability was equal to high invasion risk and low suitability was equal to low invasion risk. Except, of course, the place of origin (**Figure 1**, green dots) (Issaly et al., 2024). The raster file was converted into a surface file using the “From Raster” function in the ArcGIS conversion tool. Then, the calculation function in the ArcGIS attributes was used to calculate the area of each invasion threat area.

## 3 Results

### 3.1 Accuracy of the MaxEnt prediction model

**Figure 2:**
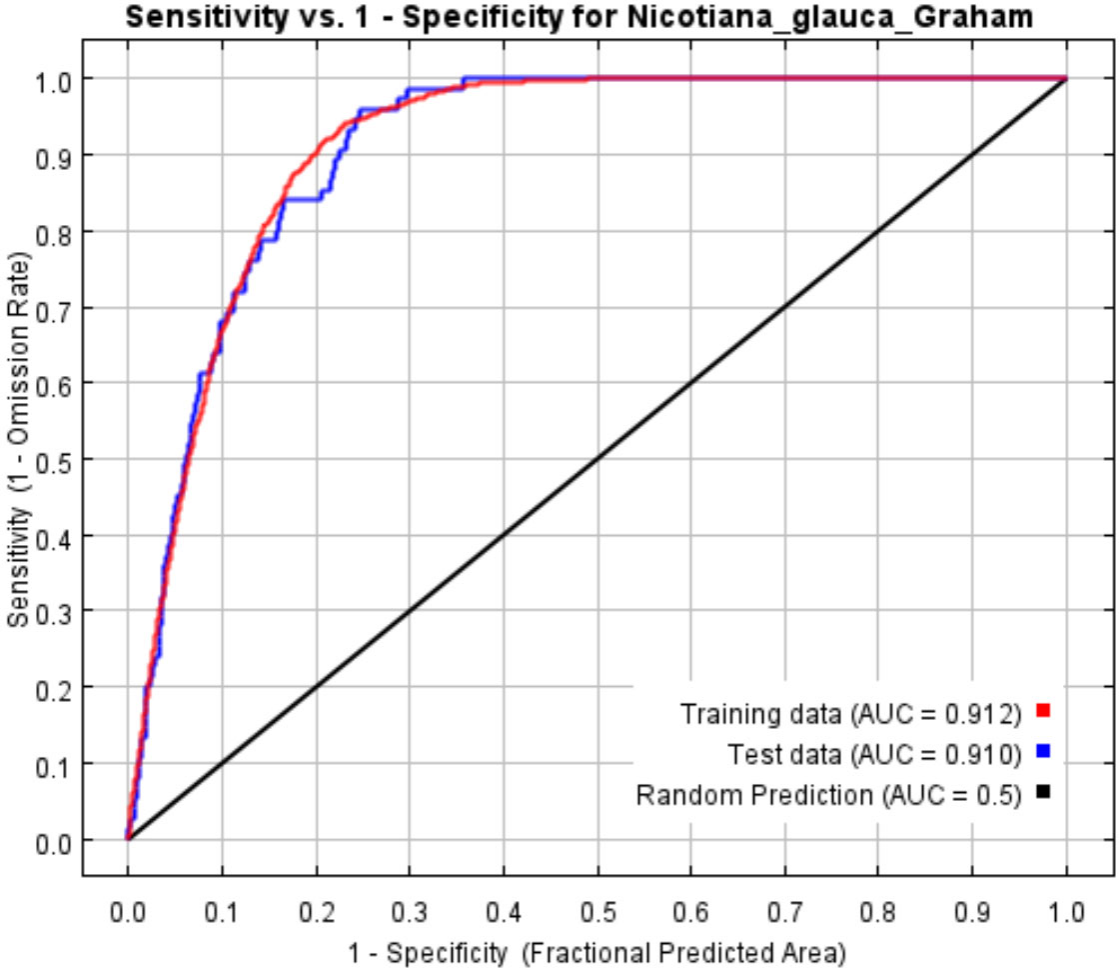
ROC curve and AUC of the MaxEnt model.

In order to determine the accuracy of the MaxEnt prediction model, the ROC curve and the corresponding AUC values, as well as the CBI, TSS and Kappa values, were calculated. The results showed that (**Figure 2**), the AUC values for the test data (0.910) and training data (0.912) were both greater than 0.9, indicating excellent discrimination ability of the MaxEnt prediction model. The TSS was 0.753, suggesting good predictive performance. The CBI reached 0.994, demonstrating a strong agreement between predicted suitability and the distribution of occurrence points. The Kappa value was 0.380, which was moderate and partly reflected the influence of imbalanced presence-background data and threshold selection. The consistent results of AUC, TSS, and CBI indicated that the model exhibited excellent discriminatory ability and predictive accuracy.

### 3.2 Influence of each bioclimatic variable on the construction of the MaxEnt prediction model

In order to characterize the influence of each bioclimatic variable on the construction of the MaxEnt prediction model, the contribution rate of each of the six bioclimatic variables to the construction of the MaxEnt prediction model for the prediction of the *N. glauca*’s global suitable habitats was calculated. The results showed that, among the six bioclimatic variables used to construct the MaxEnt prediction model (**Table 3**), the top three contributors were isothermality (bio3, 55.2%), the maximum temperature of the warmest month (bio5, 31.3%), and the average temperature of the wettest season (bio8, 6.0%), together accounting for 92.5% of the predictive power. The remaining three bioclimatic variables, precipitation of warmest quarter (bio18, 3.9%), mean diurnal range (bio2, 2.8%), and precipitation seasonality (bio15, 0.8%), contributed only 7.5% in total. These results indicated that isothermality (bio3, 55.2%), the maximum temperature of the warmest month (bio5, 31.3%), and the average temperature of the wettest season (bio8, 6.0%) were the main bioclimatic variables that affected the results of the MaxEnt prediction model construction.

**Table 3:** Contribution of the six bioclimatic variables to the construction of the MaxEnt model.

Bioclimatic variables	Contribution rate (%)
Isothermality (bio3)	55.2
Max Temperature of Warmest Month (bio5)	31.3
Mean Temperature of Wettest Quarter (bio8)	6.0
Precipitation of Warmest Quarter (bio18)	3.9
Mean Diurnal Range (bio2)	2.8
Precipitation Seasonality (bio15)	0.8

### 3.3 Key bioclimatic variables affecting prediction results

**Figure 3:**
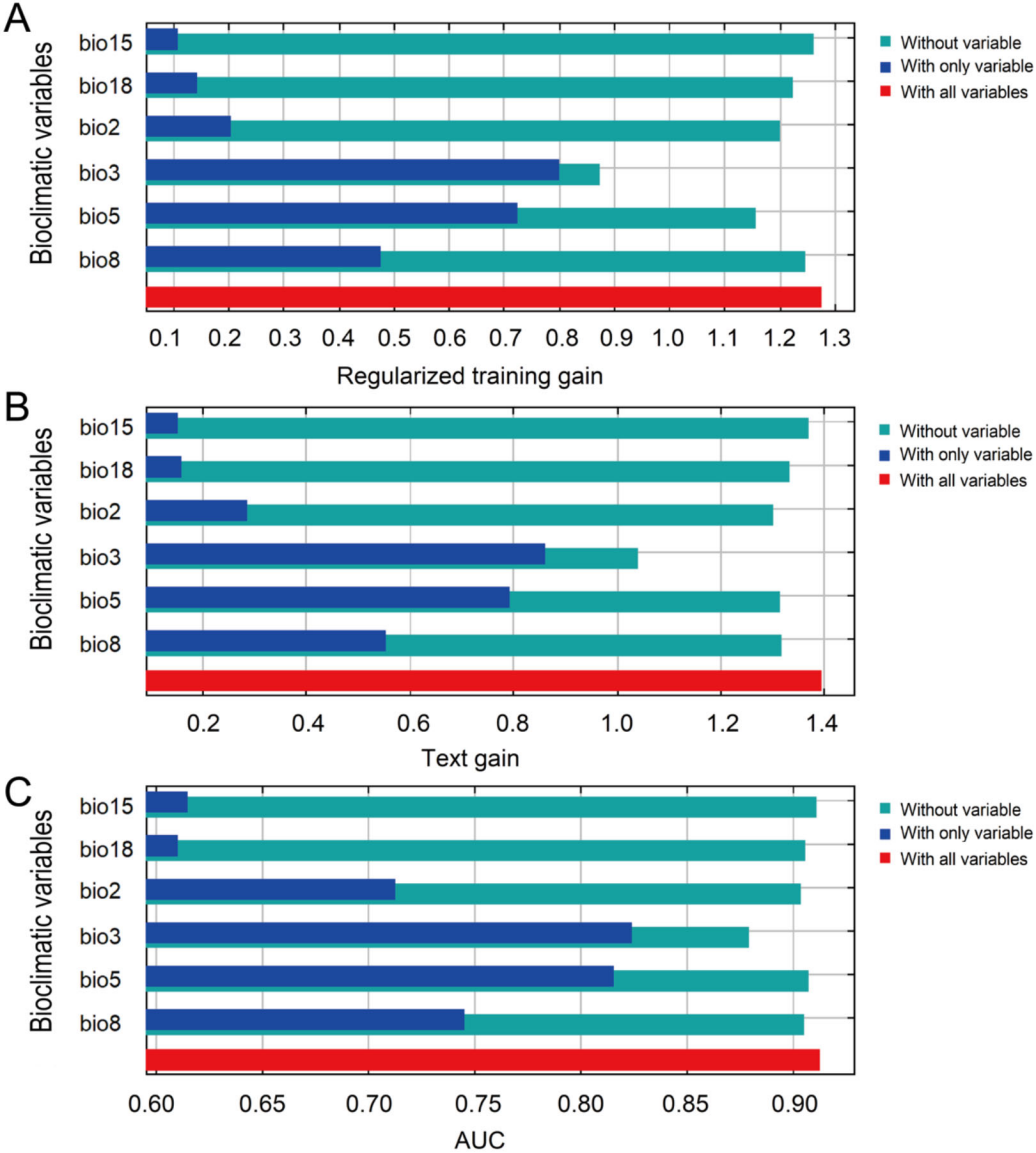
The jackknife test of the MaxEnt model. **(A)** The regularized training gain. **(B)** The text gain. **(C)** The AUC.

In order to characterize the impact of each bioclimatic variable on the model’s predictions, each bioclimatic variable used to build the MaxEnt prediction model was further tested using the jackknife test (**Figure 3**). For the blue bar diagram, its length is positively correlated with the importance of the variable. The results of the blue bar diagram showed that bio3, bio5, bio8, and bio2 were all the most important four bioclimatic variables among the regularized training gain (**Figure 3A**), test gain (**Figure 3B**), and AUC (**Figure 3C**). For the green bar diagram, its length is negatively correlated with the special information contained in this variable. The results of the green bar diagram showed that bio3 was the variable with the shortest bar among the three tests, suggesting that bio3 provided the most specific information in the prediction process. Overall, the results of the jackknife test indicated that bio3, bio5, bio8, and bio2 were the key bioclimatic variables affecting the prediction results of *N. glauca*’s potential invasion threat area.

**Figure 4:**
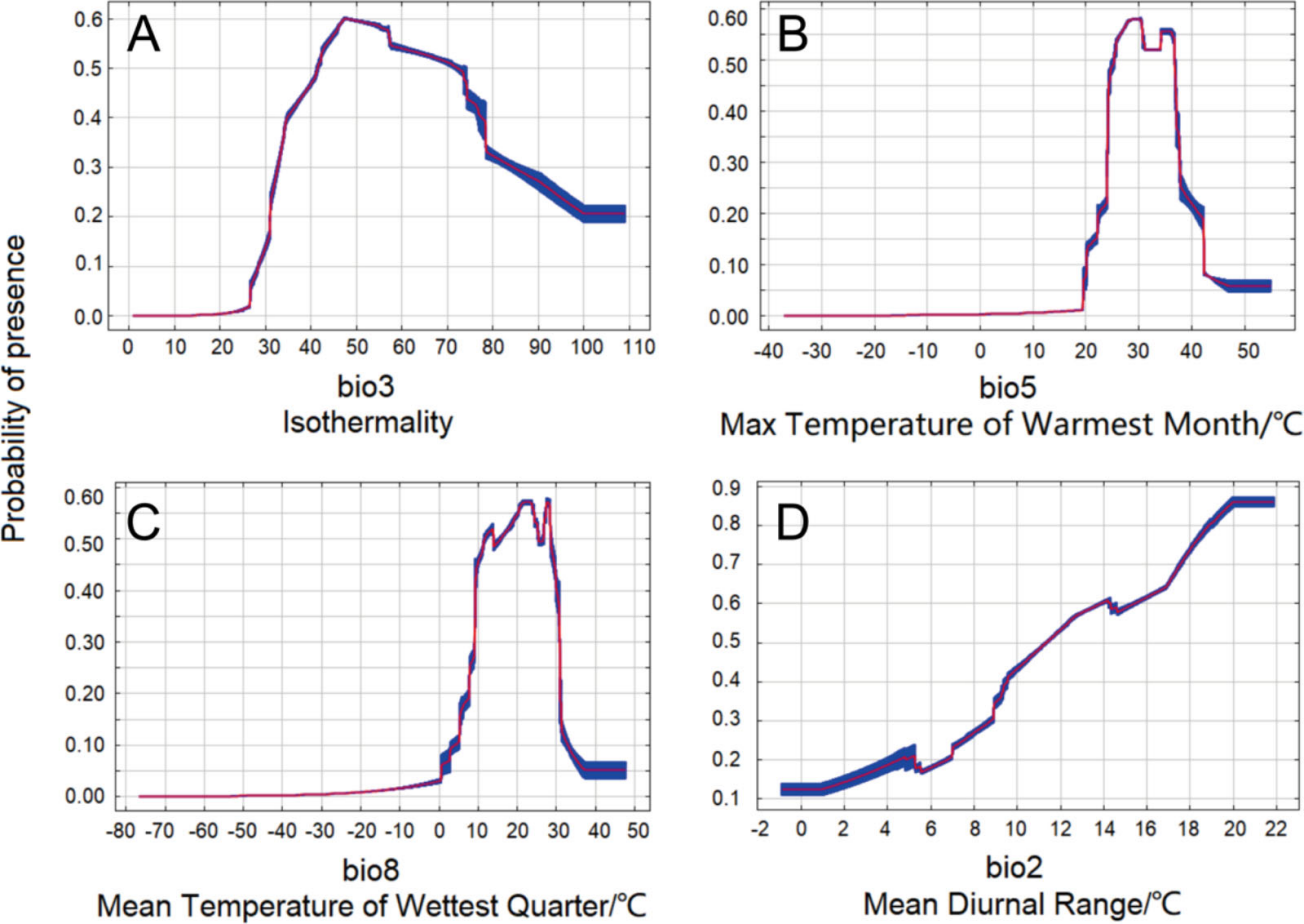
Response curves of key bioclimatic variables. **(A)** The response curve of isothermality (bio3). **(B)** The response curve of max temperature of warmest month (bio5). **(C)** The response curve of mean temperature of wettest quarter (bio8). **(D)** The response curve of mean diurnal range (bio2).

In order to reveal the value range of key bioclimatic variables, response curves of bio3, bio5, bio8, and bio2 were further calculated (**Figure 4**). The results showed that response curves of bio3, bio5, and bio8 all had a trend of first rising and then falling (**Figures 4A–C**, respectively), while the response curve of bio2 generally had a trend of continuous rise before 20°C (**Figure 4D**), which meant that bio3, bio5, and bio8 had the optimal growth range, while for bio2, before 20°C, growth increased with rising temperature, and after 20°C, the correlation tended to level off. When the presence probability was greater than 0.46, that is, in the high-threat area of *N*. *glauca*, the value range of bio3 was 40-74 (**Figure 4A**), the value range of bio5 was 25-37°C (**Figure 4B**), the value range of bio8 was 11-29°C (**Figure 4C**), and the value range of bio2 was 11-22°C (**Figure 4D**). The peaks of these response curves occurred at 48, 30°C, 28°C, and 20°C for bio3, bio5, bio8, and bio2, respectively, representing that at these value points, the presence probability of *N*. *glauca* reached the highest.

### 3.4 Potential invasion threat area under historical climate conditions

**Figure 5:**
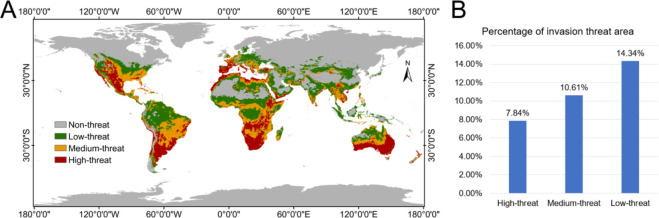
Potential invasion threat area of *N. glauca* under historical climate conditions. **(A)** Geographical distribution of different invasion threat levels. **(B)** Percentage of invasion threat area.

The prediction results showed that *N. glauca* was distributed between 60°N-45°S under historical climate conditions (**Figure 5A**). Statistically, the proportion of their areas gradually decreased from the low threat area (14.34%), medium threat area (10.61%) to high threat area (7.84%) (**Figure 5B**). The high threat area of *N. glauca* were found in the eastern and southern coastal areas of Oceania, the pampas grasslands and the eastern and western coastal areas of South America, the Kalahadi Basin and its northern region of southern Africa, the Mediterranean coast, the Cordillera and the Great Plains of western North America, and the eastern region of the Central and Southern Peninsula of Asia (**Figure 5A**, red area). Most of these high-threat areas were generally concentrated in the southern coastal areas of the continents. The medium-threat area of *N. glauca* was found in the Central and Southeast coast of North America, Central South America, Central and Northern interior of Africa, Central Europe, Southern coast of Asia, and Central Oceania (**Figure 5A**, orange area). Most of these medium-threat areas were located to the north of the high-threat area or the area extending inland to the east or west of the high-threat area. The low threat area of *N. glauca* was found in north-central North America, north-central South America, north-central Africa, Central Europe, north-central Asia, and north-central Oceania (**Figure 5A**, green area). These low-threat areas were generally located to the north of the medium-threat area or the area extending inland adjacent to the medium-threat area. In general, the threat areas were distributed in layers or clumps on each continent, and the degree of the threat decreased gradually from south latitude to north latitude.

In China, *N. glauca* has a wide range of invasion threat areas (**Figure 5A**). The high invasion threat area of *N. glauca* was concentrated in most parts of Hainan and Yunnan provinces and a little part of Qinghai, Xinjiang, Fujian and Guangdong provinces. The medium threat area of *N. glauca* was distributed in the southern part of Xinjiang, Inner Mongolia and Sichuan provinces, the northern part of Qinghai and Gansu provinces, the south-western part of Guizhou province, the eastern part of Guangxi province, and most parts of Fujian and Taiwan provinces. The low invasion threat area of *N. glauca* were distributed in China’s central and western regions and some southern provinces, including the south-central part of Xinjiang province, the northern and southern parts of Gansu province, Ningxia province, the southwestern parts of Inner Mongolia province, Shaanxi province, Shanxi province, Hebei province, Henan province, the central and southwestern parts of Shandong province, Anhui province, the northwestern parts of Hubei province, the northeastern parts of Sichuan province, the northeastern parts of Guizhou, Guangxi province, Guangdong province, Fujian province, the southern parts of Zhejiang province, etc. In high-invasion-threat areas of *N. glauca* in China, preventive and control measures can be implemented, including intercepting its seeds and seedlings through inspections of inbound logistics, and setting up monitoring sites in invasion-prone areas for early warning and surveillance.

### 3.5 Changes of the potential invasion threat area under future climate conditions

**Figure 6:**
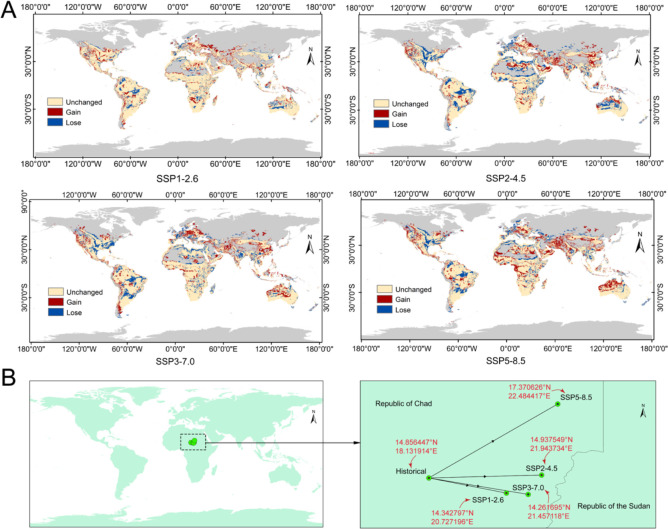
Changes in invasion threat area of *N. glauca* under different climate scenarios in the future. **(A)** regional changes of the invasion threat area. **(B)** migration of the center of the mass of the invasion threat area.

Based on the prediction results of the MaxEnt model, the distribution areas of the potentially suitable habitats of *N. glauca* worldwide under the SSP1-2.6, SSP2-4.5, SSP3-7.0, and SSP5-8.5 scenarios during the 2061s-2080s were obtained (**Figure 6A**). There were no significant changes in the suitable habitats of *N. glauca* under the SSP1-2.6 scenario in South America, the United States, Australia, central-southern Africa, most parts of Europe, and some regions of China (**Figure 6A**, the upper left panel). Under the SSP2-4.5 (**Figure 6A**, the upper right panel), SSP3-7.0 (**Figure 6A**, the lower left panel), and SSP5-8.5 (**Figure 6A**, the lower right panel) scenarios, the regions with reduced invasion of *N. glauca* were mainly concentrated in the east-central part of North America and around 60°W in South America. Across all four climate scenarios, the newly increased suitable habitats of *N. glauca* were predominantly distributed in the western coastal zones of the Americas, coastal areas of Africa, China, central Europe, the Arabian region, and most parts of Australia. In China, the newly expanded invasive areas were mainly concentrated in parts of the southwest, northwest, and southeast regions. Particularly under the SSP3-7.0 and SSP5-8.5 scenarios, the newly expanded areas were the largest in scale and the highest in threat level, with contiguous high-threat areas appearing in most parts of Australia, the western coastal zones of the Americas, and central Europe. Migration of the center of the mass of the invasion threat area (**Figure 6B**) showed that the centroid of *N. glauca*’s invasion threat area presented a consistent shift toward the southeast and northeast migration trend relative to the present period under all four future climate scenarios. Among the scenarios, the migration distance is the longest under the high-emission SSP5-8.5 scenario (from 14.856447°N and 18.131914°E to 17.370626°N and 22.484417°E), followed by the SSP2-4.5 scenario (from 14.856447°N and 18.131914°E to 14.937549°N and 21.943734°E); in contrast, the migration distance is significantly shorter under the low-emission SSP1-2.6 scenario (from 14.856447°N and 18.131914°E to 14.342797°N and 20.727196°E) and the medium-emission SSP3-7.0 scenario (from 14.856447°N and 18.131914°E to 14.261695°N and 21.457118°E), this may be due to the limited temperature rise, resulting in a limited expansion of the suitable habitat; and the medium−emission SSP3-7.0 scenario leads to a higher temperature increase, the precipitation changes are complex, which may partially offset the expansion caused. In **Figure 6B**, the center of the mass showed an overall eastward shift. Under the high−emission SSP5−8.5 scenario, the center of the mass shifted markedly eastward and northward. From a topographic perspective, this shift is likely caused by the Ennedi Plateau located at the border between eastern/northern Chad and Sudan. Global warming has expanded suitable habitats at higher latitudes and altitudes. The lack of a direct northward shift may be due to the Sahara Desert in the northern region, where extreme aridity limits the survival of *N. glauca*. The resulting data can provide future early warnings for regions where the suitable habitats of *N. glauca* will expand under different climatic conditions.

The distribution of suitable habitats for *N. glauca* across threat levels varies under different climate scenarios (**Table 4**). The proportion of low-threat areas was highest under SSP3-7.0 and lowest under SSP5-8.5. Notably, only under SSP5-8.5 did this proportion fall below that of the historical scenario, suggesting a general expansion of low-threat habitats in future climates. In contrast, the proportion of medium-threat areas was consistently higher under all future scenarios compared to the historical baseline, indicating a continued enlargement of such regions. The share of high-threat areas across scenarios followed the order: SSP1-2.6 > SSP5-8.5 > historical > SSP3-7.0 > SSP2-4.5, reflecting that the extent of high-threat habitats may either increase or decrease depending on the specific climate pathway. Overall, the total threatened area was larger in all future scenarios than in the historical reference, collectively pointing to an expanding trend of threatened habitats under future climate conditions.

**Table 4:** Area proportions of suitable habitat areas under different scenarios.

Threat level	SSP1-2.6	SSP2-4.5	SSP3-7.0	SSP5-8.5	Historical
Low-threat	15.01%	14.49%	15.26%	14.21%	14.34%
Medium-threat	11.28%	11.74%	11.57%	12.20%	10.61%
High-threat	8.34%	7.46%	7.82%	8.29%	7.84%
Total	34.63%	33.70%	34.65%	34.70%	32.79%
Compared to historical	+1.84%	+0.91%	+1.87%	+1.91%	0

## 4 Discussion

*N. glauca* is an invasive species with both utilization value and hazardous properties, showing a trend of local expansion globally. Comparing it with other typical invasive species can further clarify its invasive potential and provide more targeted theoretical basis for cross-species invasion prevention and control strategies. Climatic factors (bio3: Isothermality; bio5: Max Temperature of Warmest Month; bio8: Mean Temperature of Wettest Quarter; bio2: Mean Diurnal Range) are the dominant factors determining the distribution of *N. glauca*. This temperature adaptation pattern has both commonalities and significant differences with invasive plants of the same *Solanaceae* family and other families. Taking invasive species of the same *Solanaceae* family, such as *Solanum aculeatissimum* and *Solanum viarum* Dunal, as examples: human pressure and precipitation of the warmest quarter(bio18) are the most critical predictive factors for them, respectively. The two species have similar suitable temperature ranges (15-20 °C) and largely overlapping main altitude distribution areas (800–1959 m) (Jan et al., 2025; Sun and Deng, 2025). This similarity may be attributed to their close genetic relationship as members of the *Solanum* genus. In contrast, although all three species are native to South America, *N. glauca* has a wider high-temperature tolerance range (7-35 °C) (Florentine et al., 2016) (bio5, 25-37 °C in this study), a higher altitude distribution range (0–3600 m) (Gonzalez et al., 2012), and achieves the highest germination rate under a day-night temperature difference of 20 °C/15 °C (Brandes, 2001). This difference may result from the evolutionary adaptation of *N. glauca* to mountainous and other environments.

Compared with non-*Solanaceae* invasive weeds, the difference in precipitation adaptability between *N. glauca* and the moisture-loving invasive species *Spartina alterniflora* Loisel is particularly significant. *Spartina alterniflora* is mainly affected by mean diurnal range (bio2) and precipitation variables (bio14, bio19), with salt and waterlogging tolerance, and is especially adapted to the tidal environment along the southern coast of China (Liu et al., 2025). In contrast, *N. glauca* has a wider tolerance range for precipitation in the warmest quarter and can distribute in arid wastelands and semi-humid riverbanks. This water adaptation pattern is similar to that of the invasive species *Emex australis* (*Polygonaceae*) (Quan et al., 2023). Notably, the adaptive performance of *N. glauca* under warm and humid conditions (characterized by bio5 and bio8) does not conflict with its drought tolerance. This ecological trade-off is a key adaptive strategy for its widespread invasion. The warm and humid conditions during the growing season (bio5: 25-37 °C; bio8: 11-29 °C in high-threat areas) help to improve photosynthetic efficiency, promote nutrient absorption, and increase seed production, laying the foundation for population expansion. At the same time, the cuticular wax layer of *N. glauca* reduces water transpiration (Negin et al., 2023), enabling it to retain water during drought periods and survive in arid wastelands. This dual adaptability enhances *N. glauca*’s ability to survive in both warm and humid environments as well as arid environments.

As economic and ornamental plants belonging to the *Nicotiana* genus (*Solanaceae* family), *Nicotiana tabacum* L. (tobacco, economic crop) and *Nicotiana alata* Link et Otto (*N. alata*, ornamental plant) exhibit significant differences in their suitable habitat characteristics compared with *N. glauca*. For *Nicotiana tabacum* L., bio10 (mean temperature of the warmest quarter) and bio12 (annual precipitation) are the environmental variables with relatively strong influences on its distribution; in contrast, for *N. alata*, the key influencing environmental variables are bio9 (mean temperature of the driest quarter) and bio14 (precipitation of the driest month) (Jia et al., 2024; Zhang et al., 2023).

This discrepancy in dominant environmental factors results in distinct main distribution areas among the three species. The main suitable habitats of *Nicotiana tabacum* L. are concentrated in traditional tobacco-growing regions, such as temperate warm-season areas, including Brazil (the Americas) and Zimbabwe (Africa). *N. glauca* can produce over 1.3 million seeds per plant annually, which can be dispersed by wind and water. In the PISF region of Brazil (Nacional, 2004), *N. glauca* is densely distributed, forming a single dominant population through strong allelopathic effects (Fabricante et al., 2015). For *N. alata*, its distribution is primarily in regions with temperate to subtropical mild climates, such as Europe and southeastern South America (Jia et al., 2024; Zhang et al., 2023). With global climate warming, the core suitable habitats of both *Nicotiana tabacum* L. and *N. alata* exhibit the characteristic of expanding toward higher latitudes. As non-invasive plants, neither of them possesses active invasive ability, and their distributions are significantly influenced by human cultivation activities.

Under future climate scenarios (2061-2080), the global suitable distribution area of *N. glauca* presents the characteristics of “stable core areas and local expansion”: there is no significant change in core distribution areas such as southern South America and central-southern Africa, while new suitable areas emerge in the western coastal areas of the Americas, central Europe, and most parts of Australia. Moreover, the expansion is more obvious under high-emission scenarios (SSP3-7.0, SSP5-8.5). This invasive trend shares commonalities with the “northward expansion” characteristic of most warm-loving invasive species but differs in the scope and process of expansion. Taking four invasive *Amaranthaceae* species (*Dysphania ambrosioides*, *Celosia argentea*, *Amaranthus palmeri*, and *Amaranthus spinosus*) as examples, with the trend of climate warming, the distribution centers of all four species have shifted northward. Under the SSP126 scenario, *A. palmeri* has the largest expansion range and continues to expand together with *A. spinosus*, while *D. ambrosioides* and *C. argentea* undergo an invasive process of initial contraction followed by expansion (Lin et al., 2025).

This study only selected climatic variables to construct the prediction model. However, in other research, non-climatic factors, such as topographic variables (including slope and aspect), the Human Influence Index (HII), which integrates population density, land use and cover, transportation networks, and light pollution, soil properties, and even animal activities, are often used to construct prediction models or have even been verified as key factors affecting the invasion. In the research on the global invasion potential of *Bidens Pilosa* (*Asteraceae*), it was found that the human footprint is one of the core factors affecting its distribution (Fan et al., 2025). The increasingly frequent maritime trade has enabled it to spread from its native tropical America to more than 40 countries around the world (Fan et al., 2025). Some studies have found that rivers, particularly urban rivers or urban streams, serve as important dispersal corridors for the invasion and spread of alien species (Francis et al., 2019). Roads have also been identified as corridors for invasive plants (Adhikari et al., 2024). And another study has shown that animals may serve as vectors for secondary dispersal in some regions (Chuong et al., 2016; Seefeldt et al., 2010). On the other hand, unevenly distributed sample points are also a limitation of the model input data in this study. Although we applied spatial thinning to reduce sampling bias, the occurrence records of *N. glauca* remain unevenly distributed across regions, with dense sampling in South America (its native range) and sparse sampling in Asia (where it is cultivated or naturalized to a lesser extent). This geographical imbalance may lead to two main limitations. First, the MaxEnt model could overfit to environmental conditions prevalent in South America, potentially underestimating or overestimating suitability in data-poor regions such as Asia. Second, the predictive accuracy in regions with few sample points may be reduced, which is partly reflected in the moderate Kappa value (0.38), even though other metrics (AUC>0.91, TSS = 0.75, CBI = 0.994) indicate strong overall model performance. Nevertheless, the consistently high AUC, TSS, and CBI suggest that the model captures the core ecological niche of *N. glauca* reliably. Therefore, future studies should aim to collect more occurrence data from Asia and other regions with few sample points, and consider using geographically stratified cross-validation or ensemble modelling to further improve model transferability. Taken together, in future research, more non-climatic factors and more evenly distributed sample points should be incorporated to enhance the quality of the study.

In this study, we approximately regarded the suitability degree as the degree of invasion risk, and simply believed that high suitability was equal to high invasion risk. However, this is an idealized assumption. In the actual ecological environment, the risk of invasion also needs to take into account the ecological stability and species richness of the area itself. A study on invasion risk in Badong County, Hubei Province, China, revealed a significant spatial negative correlation between comprehensive invasion risk and habitat quality. Regions with high habitat quality exhibit strong resistance to invasion due to their stable ecosystems and high species richness (Wu et al., 2025). Regarding the role of resource competition in biological invasions, another study has shown that competition among invasive species can lead to excessive consumption of growth resources, thereby restricting their reproduction (Gioria and Osborne, 2014). Furthermore, such competition can also degrade habitat quality (Ehrenfeld, 2010). Therefore, the invasion risk areas predicted in this study still need to be tested in practice. To obtain accurate prediction results of invasion risk areas, factors such as species richness, ecosystem stability and species competition need to be introduced.

Our study focused on predicting global potentially suitable habitats and assessing early invasion risk for *N. glauca*, without conducting an in−depth analysis of niche dynamics. In contrast, Issaly combined phylogeographic analysis (chloroplast DNA haplotypes) with ecological niche modeling using ellipsoid models based on Mahalanobis distance to trace the invasion origin of this species (Issaly et al., 2024). Their results showed that only one native genetic lineage contributed to the invasion, yet no climatic niche overlap existed between this native source and any invaded area. Instead, a bridgehead population in western South America exhibited higher genetic diversity and a shift toward drier, colder climates, and this population showed climatic overlap with most other invaded regions. The authors thus proposed that *N. glauca* first invaded western South America, underwent an environmental niche shift, and subsequently spread worldwide from this bridgehead. The ellipsoid modeling approach used by Issaly requires fewer occurrence points and is mathematically transparent, making it robust for comparing niches among small genetic lineages. However, it assumes multivariate normality and cannot capture complex, non−convex environmental responses. Conversely, MaxEnt handles non−linear interactions, feature selection, and regularization, making it more powerful for predicting global distributions, albeit with a higher demand for occurrence data and greater sensitivity to background sampling. Given these complementary strengths, MaxEnt can also be applied to study niche dynamics of *N. glauca* (including niche shift, expansion, and conservatism) in the future, allowing a comparative discussion with previous findings. It should be noted that for niche dynamics studies, MaxEnt is often combined with background−correction methods such as PCA−env rather than used alone. Overall, for strict lineage−level niche comparisons, simpler models like the ellipsoid are preferable, whereas MaxEnt is more suitable for global invasion predictions.

Finally, for the highly suitable areas of *N. glauca*, that is, the regions approximately regarded as having a high risk of invasion in this study, such as Hainan, Yunnan, southern Fujian and Guangdong in southern China, Australia, and South America, the following targeted and operable monitoring and management strategies were proposed. First, a cross-provincial joint monitoring system should be established, with fixed monitoring points set up in invasion-prone habitats (including riverbanks, wastelands, and edges of farmland), and drone patrols and ground surveys employed to track invasion dynamics in real time. Second, strict quarantine inspections should be implemented on inbound logistics and seedling trade in border areas and coastal ports to intercept *N. glauca* seeds and seedlings. Third, for concentrated distribution areas, intensive manual eradication should be carried out before the annual flowering period. Fourth, regional ecological restoration can be achieved by planting local plants, improving the structure of local plant communities, enhancing species diversity, and strengthening resistance to *N. glauca* invasion. Additionally, appropriate interference or disruption measures can be adopted, targeting factors that promote the growth of *N. glauca*. Such as the application of Actinobacteria inhibitors, since the invasion of *N. glauca* in semiarid regions is achieved by selectively enriching functional microorganisms (e.g., Actinobacteria) to construct a soil environment suitable for its survival (Caravaca et al., 2024).

## Data Availability

Publicly available datasets were analyzed in this study. This data can be found here: GBIF, https://www.GBIF.org.

## References

[B1] Adhikari A. Subedi A. Tiwari A. Shrestha B. B. (2024). Impacts of road on plant invasions in the middle mountain region of central Nepal. J. Mountain Sci. 21, 619–632. doi: 10.1007/s11629-023-8064-z. PMID: 30311153

[B2] Adhikari P. Lee Y. H. Adhikari P. Poudel A. Seo C. Lee D.-H. . (2025). Global assessment of invasion risk: Ardisia elliptica, one of the most noxious tropical shrubs in the world. Ecol. Processes 14, 55. doi: 10.1186/s13717-025-00622-z. PMID: 38164791

[B3] Assaeed A. M. Alharthi A. S. Abd-Elgawad A. M. (2021). Impacts of Nicotiana glauca Graham invasion on the vegetation composition and soil: A case study of Taif, Western Saudi Arabia. Plants (Basel) 10, 2587. doi: 10.3390/plants10122587. PMID: 34961058 PMC8708854

[B4] BCC (2019). WCRP CMIP6: Beijing Climate Center (BCC) BCC-CSM2-MR model output for the "hist-aer" experiment (United Kingdom: CEDA). C. F. E. D. A. (ed.).

[B5] Brandes D. (2001). Nicotiana glauca als invasive Pflanze auf Fuerteventura. Braunschweiger Geobotanische Arbeiten 8, 39–57.

[B6] Cao J. Xu J. Pan X. Monaco T. A. Zhao K. Wang D. . (2021). Potential impact of climate change on the global geographical distribution of the invasive species, Cenchrus spinifex (Field sandbur, Gramineae). Ecol. Indic. 131, 108204. doi: 10.1016/j.ecolind.2021.108204. PMID: 38826717

[B7] Caravaca F. Torres P. Díaz G. Roldán A. (2024). Selective shifts in the rhizosphere microbiome during the drought season could explain the success of the invader Nicotiana glauca in semiarid ecosystems. Sci. Total Environ. 946, 174444. doi: 10.2139/ssrn.4772507 38964394

[B8] Chuong J. Huxley J. Spotswood E. N. Nichols L. Mariotte P. Suding K. N. (2016). Cattle as dispersal vectors of invasive and introduced plants in a California annual grassland. Rangel. Ecol. Manag. 69, 52–58. doi: 10.1016/j.rama.2015.10.009

[B9] Cronk Q. C. B. Fuller J. L. (2001). Plant invaders: the threat to natural ecosystems (London: Routledge).

[B10] Cui Y. Ni X. Jiang Z. Song Y. Bao X. (2025). Analysis of influencing factors and trend prediction of invasive alien plants in China. Diversity 17, 521. doi: 10.3390/d17080521. PMID: 30654563

[B11] Dounas H. Bouskout M. Nafidi H.-A. Alsahli A. A. Bourhia M. Ouahmane L. (2023). Unraveling arbuscular mycorrhizal fungi interactions in the exotic plant Nicotiana glauca Graham for enhanced soil fertility and alleviation of metal pollution. Horticulturae 9, 585. doi: 10.3390/horticulturae9050585. PMID: 30654563

[B12] Ehrenfeld J. G. (2010). Ecosystem consequences of biological invasions. Annu. Rev. Ecol. Evol. Syst. 41, 59–80. doi: 10.1146/annurev-ecolsys-102209-144650. PMID: 41139587

[B13] Fabricante J. R. De Castro R. A. De Araújo K. C. T. De Siqueira J. A. (2015). Ecological attributes of alien Nicotiana glauca graham (solanaceae) and assessment of the susceptibility of the species occurring in Brazil. Cienc. Florestal 25, 959–967. doi: 10.5902/1980509820650

[B14] Fan L. Mi C. Li J. Zhang Y. Zhang H. Zhang G. . (2025). Projecting global shifts in the invasive potential of Bidens pilosa L. under climate change using species distribution models. Front. Plant Sci. 16. doi: 10.3389/fpls.2025.1580278. PMID: 40443437 PMC12119604

[B15] Florentine S. Weller S. Graz F. P. Westbrooke M. Florentine A. Javaid M. . (2016). Influence of selected environmental factors on seed germination and seedling survival of the arid zone invasive species tobacco bush (Nicotiana glauca R. Graham). Rangeland J. 38, 417–425. doi: 10.1071/rj16022. PMID: 38477348

[B16] Francis R. A. Chadwick M. A. Turbelin A. J. (2019). An overview of non‐native species invasions in urban river corridors. River Res. Appl. 35, 1269–1278. doi: 10.1002/rra.3513. PMID: 41531421

[B17] Furer V. Hersch M. Silvetzki N. Breuer G. S. Zevin S. (2011). Nicotiana glauca (tree tobacco) intoxication--two cases in one family. J. Med. Toxicol. 7, 47–51. doi: 10.1007/s13181-010-0102-x. PMID: 20652661 PMC3614112

[B18] Gioria M. Osborne B. A. (2014). Resource competition in plant invasions: emerging patterns and research needs. Front. Plant Sci. 5. doi: 10.3389/fpls.2014.00501. PMID: 25324851 PMC4179379

[B19] Gonzalez A. Tezara W. Rengifo E. Herrera A. (2012). Ecophysiological responses to drought and salinity in the cosmopolitan invader Nicotiana glauca. Braz. J. Plant Physiol. 24, 213–222. doi: 10.1590/S1677-04202012000300008

[B20] Huang T. Yang T. Wang K. Huang W. (2024). Assessing the current and future potential distribution of Solanum rostratum Dunal in China using multisource remote sensing data and principal component analysis. Remote Sens. 16, 271. doi: 10.3390/rs16020271. PMID: 30654563

[B21] Issaly E. A. Baranzelli M. C. Rocamundi N. Ferreiro A. M. Johnson L. A. Sérsic A. N. . (2024). Too much water under the bridge: unraveling the worldwide invasion of the tree tobacco through genetic and ecological approaches. Biol. Invasions 26, 515–533. doi: 10.1007/s10530-023-03189-y. PMID: 30311153

[B22] Jan S. Mishra A. K. Shah S. A. Bhat M. A. Wani Z. A. Jan A. T. (2025). Distribution pattern and habitat suitability modelling of an invasive plant species – Solanum viarum Dunal. J. Nat. Conserv. 88, 127021. doi: 10.1016/j.jnc.2025.127021. PMID: 38826717

[B23] Jia L. Sun M. He M. Yang M. Zhang M. Yu H. (2024). Study on the change of global ecological distribution of Nicotiana tabacum L. based on MaxEnt model. Front. Plant Sci. 15. doi: 10.3389/fpls.2024.1371998. PMID: 39091317 PMC11292735

[B24] Lin M. Ye X. Zhao Z. Chen S. Liu B. (2025). Comparative analysis of habitat expansion mechanisms for four invasive Amaranthaceae plants under current and future climates using MaxEnt. Plants (Basel) 14, 2363. doi: 10.3390/plants14152363. PMID: 40805712 PMC12348541

[B25] Liu W. Tao Y. He P. Liu J. Zhang W. (2025). Assessing the impacts of climate change on suitable distribution areas and ecological risks of the invasive grass (Spartina alterniflora) in China. J. Nat. Conserv. 87, 126985. doi: 10.1016/j.jnc.2025.126985. PMID: 38826717

[B26] MIN . Projeto de Integração do Rio São Francisco com Bacias Hidrográficas do Nordeste Setentrional. Relatório de Impacto Ambiental (RIMA). Brasília: Ministério da Integração Nacional, 2004 136 p.

[B27] Negin B. Hen-Avivi S. Almekias-Siegl E. Shachar L. Jander G. Aharoni A. (2023). Tree tobacco (Nicotiana glauca) cuticular wax composition is essential for leaf retention during drought, facilitating a speedy recovery following rewatering. New Phytol. 237, 1574–1589. doi: 10.1111/nph.18615. PMID: 36369885

[B28] Nyarko G. Bayor H. (2018). The effect of spatial thinning on the potential distribution of 10 African indigenous vegetables. Environ. Sci. 5, 2026–5336. doi: 10.47740/305.UDSIJD6i

[B29] Ollerton J. Watts S. Connerty S. Lock J. Parker L. Wilson I. . (2012). Pollination ecology of the invasive tree tobacco Nicotiana glauca: comparisons across native and non-native ranges. J. Pollination Ecol. 9, 85–95. doi: 10.26786/1920-7603(2012)12

[B30] Quan Z. Xuezhen Z. Lijuan Z. (2023). Prediction of potential suitable region for Emex australis in China based on the optimized MaxEnt model. J. South. China Agric. Univ. 44, 254–262. doi: 10.7671/j.issn.1001-411X.202203041

[B31] Richardson D. M. Pysek P. Rejmanek M. Barbour M. G. Panetta F. D. West C. J. (2000). Naturalization and invasion of alien plants: concepts and definitions. Divers. Distrib. 6, 93–107. doi: 10.1046/j.1472-4642.2000.00083.x. PMID: 37945311

[B32] Robert J. Henry D. R. William J. (2010). The Edinburgh New Philosophical Journal: Exhibiting a View of the Progressive Discoveries and Improvements in the Sciences and the Arts (London, UK: Nabu Press).

[B33] Rodríguez-Caballero G. Roldán A. Caravaca F. (2020). Invasive Nicotiana glauca shifts the soil microbial community composition and functioning of harsh and disturbed semiarid Mediterranean environments. Biol. Invasions 22, 2923–2940. doi: 10.1007/s10530-020-02299-1. PMID: 30311153

[B34] Sandhya Kiran G. Prajapati P. C. Mohanta A. (2024). A systematic appraisal of ecological niche modelling in the context of phytodiversity conservation. Environ. Dev. Sustainability 28, 739–769. doi: 10.1007/s10668-024-04994-8. PMID: 30311153

[B35] Seefeldt S. S. Collins W. B. Kuhl J. C. Clauss M. (2010). White sweetclover (*Melilotus albus*) and narrowleaf hawksbeard (*Crepis tectorum*) seed germination after passing through moose. Invasive Plant Sci. Manag. 3, 26–31. doi: 10.1614/IPSM-09-034.1

[B36] Sun S. Deng Z. (2025). Analysis of a potentially suitable habitat for Solanum aculeatissimum in Southwest China under climate change scenarios. Plants (Basel) 14, 1979. doi: 10.3390/plants14131979. PMID: 40647990 PMC12251579

[B37] Wu S. Chen J. Jiang S. Zhang R. Li Z. Wang L. . (2025). Invasion risk of typical invasive alien plants in mountainous areas and their interrelationship with habitat quality: A case study of Badong County in central China. J. Environ. Manage. 380, 125083. doi: 10.2139/ssrn.5052847 40157205

[B38] Wu T. Lu Y. Fang Y. Xin X. Li L. Li W. (2019). The Beijing Climate Center Climate System Model (BCC-CSM): the main progress from CMIP5 to CMIP6. Geosci. Model Dev. 12, 1573–1600. doi: 10.5194/gmd-12-1573-2019

[B39] Yuan Y. Tang X. Liu M. Liu X. Tao J. (2021). Species distribution models of the Spartina alterniflora Loisel in its origin and invasive country reveal an ecological niche shift. Front. Plant Sci. 12. doi: 10.3389/fpls.2021.738769. PMID: 34712259 PMC8546191

[B40] Zhang Y.-F. Chen S.-T. Gao Y. Yang L. Yu H. (2023). Prediction of global potential suitable habitats of Nicotiana alata Link et Otto based on MaxEnt model. Sci. Rep. 13, 4851. doi: 10.1038/s41598-023-29678-7. PMID: 36964182 PMC10038996

[B41] Zhang X. Zhao J. Wang M. Li Z. Lin S. Chen H. (2022). Potential distribution prediction of Amaranthus palmeri S. Watson in China under current and future climate scenarios. Ecol. Evol. 12, e9505. doi: 10.1002/ece3.9505. PMID: 36518625 PMC9743064

